# Sulphate of Cinchonidia

**Published:** 1877-12-20

**Authors:** 


SULPHATE OF CINCHONIDIA.—We have watched with some interest the discussion which has for some time been going on touching the relative merits of the several alkaloids, and preparations of the bark. That there are valuable and distinctive properties possessed by the several articles now so prominently brought before the profession, we have no doubt. Recently, direct inquiry has been made of us as to our views of the cinchonidia. Having been supplied with samples for experiment, we have made some observations favorable to the article named. Cases of intermittent fever have yielded promptly to the remedy, used in about the same doses as with the sulphate of quinia. We have also used it to advantage combined with elixir vitriol, to equalize and restrain the circulation in cases of menorrhagia.

In gastralgia and atonic dyspepsia, combined with tinc. nux vomica, it has been found a most admirable tonic and peristaltic persuader.

In this connection, we will append the views of Dr. J. J. Knott, of this city, who has also been experimenting with the article, from samples furnished by Messrs. Powers & Weightman. He remarks:

"I first employed it amongst the freedmen, prescribing from the packages furnished. I very soon found it equal to the sulphate of quinia in every respect, and far superior in some. The affections in which I have derived more advantage in the employment of this remedy than in the employment of quinia, are dysentery, remittent fever, and a low type of mongrel fever, which has prevailed to some extent in this climate for several years. I have one patient whose system is such that he cannot bear quinia in any form that I have administered it, on account of its producing urticaria, or nettle-rash. He took the cinchonidia without any unpleasant effects whatever. In nervous females, I find the cinchonidia preferable to the quinia. For the past two years, I have used, comparatively, but little quinia in my practice, having substituted the cinchonidia for it. A favorite formula with me is the following, for an adult:

℞. Sulph. Cinchonidia, grs. xvi
Ext. Belladonna, grs $\frac{1}{4}$—$\frac{1}{2}$
Pulv. Capsicum, grs. ij
Pulv. Doveri, grs. ij
Salicylic acid, grs. iv. M.

Put into four capsules, one to be given every two hours. I sometimes add to this prescription four to six grains of calomel, when indicated. In the treatment of some affections, I regard the sulphate of cinchonidia as great an improvement over quinine as the latter is over the old remedy, the crude bark."

We publish, also, the following communication from A. G. Hobbs, M. D., of Indiana:

SULPHATE CINCHONIDIA.

I think when sul. cinchonidia becomes more generally the substitute for sul. quinia, there will be but little use of the now much-vaunted remedy, hydrobromic acid for *tinnitus aurium.* In malarious districts, such as is southern Indiana, cinchonidia is the country practitioners' greatest boon.

The difference in its cost as compared with quinia—one-fourth—is no small item to him who has his two to three ounces a week to buy.

During the last four months, I nave used cinchonidia almost exclusively in nearly three hundred cases of chills, intermittent, remittent and bilious fevers, and out of the whole number, have been compelled to resort to arsenic in but three cases of chills.

My experience with cinchonidia in these three hundred cases of malarious fevers, is as follows:

1. I think it fully equal to quinia as an anti-periodic. Have never used it exclusively as an anti-pyretic, as in typhoid fever, pneumonia, etc., but if I ever find it necessary, I shall not hesitate to risk it as such.

2. It produces no *tinnitus aurium*—at least, I have never been able to discover it in the size doses that I administer it to break malarious attacks.

3. The stomach, undoubtedly, tolerates it better than quinine

4. I find it, so far as I can observe, fully equal to quinia as a tonic, in combination with iron. I administer it in doses same as quinia by bulk, which is about one-third greater by weight.

